# Distributed Practice: Rarely Realized in Self-Regulated Mathematical Learning

**DOI:** 10.3389/fpsyg.2018.02170

**Published:** 2018-11-20

**Authors:** Katharina Barzagar Nazari, Mirjam Ebersbach

**Affiliations:** Department of Psychology, Developmental Psychology, University of Kassel, Kassel, Germany

**Keywords:** desirable difficulties, distributed practice, spacing effect, mathematics, high school students, self-regulated learning, individual differences

## Abstract

The purpose of the present study was to investigate the effect and use of distributed practice in the context of self-regulated mathematical learning in high school. With distributed practice, a fixed learning duration is spread over several sessions, whereas with massed practice, the same time is spent learning in one session. Distributed practice has been proven to be an effective tool for improving long-term retention of verbal material and simple procedural knowledge in mathematics, at least when the practice schedule is externally guided. In the present study, distributed practice was investigated in a context that required a higher degree of self-regulation. In total, 158 secondary school students were invited to participate. After motivational and cognitive characteristics of the students were assessed, the students were introduced to basic statistics, a topic of their regular curriculum. At the end of the introduction, the students could sign up for the study to further practice this content. Eighty-seven students did so and were randomly assigned either to the distributed or to the massed practice condition. In the distributed practice condition, they received three practice sets on three different days. In the massed practice condition, they received the same three sets, but all on one day. All exercises were worked in the context of self-regulated learning at home. Performance was tested 2 weeks after the last practice set. Only 44 students finished the study, which hampered the analysis of the effect of distributed practice. The characteristics of the students who completed the exercises were analyzed exploratory: The proportion of students who finished all exercises was significantly higher in the massed than in the distributed practice condition. Within the distributed practice condition, a significantly larger proportion of female students completed the exercises compared to male students. Additionally, among these female students, a larger proportion showed lower concentration difficulty. No such differential effects were revealed in the massed practice condition. Our results suggest that the use of distributed practice in the context of self-regulated learning might depend on learner characteristics. Accordingly, distributed practice might obtain more reliable effects in more externally guided learning contexts.

## Introduction

Teachers at school and university should generally be interested in learning techniques that promote long-term retention of the learned contents. The reason for this is that most topics taught in school or at university are rather complex and many advanced topics rely – sometimes more, sometimes less so – on prior knowledge. In mathematics, for example, in order to grasp stochastics, solid knowledge of fractional arithmetic is helpful. That is why in most cases it is important to use learning strategies that help to store knowledge in a way that facilitates long-term retention, rather than learning strategies that result in knowledge that can be retrieved only for a short period after it was taught and acquired.

One branch of learning strategies that promote long-term retention is based on so-called desirable difficulties (Bjork, 1994). Desirable difficulties are mechanisms that slow down the learning process and make it harder for the learner, but boost long-term performance. Learning strategies that make use of these desirable difficulties are, among others, the generation of (part of) the content that has to be learned (McDaniel et al., 1988), (self-)testing as part of the learning process (Karpicke and Roediger, 2008), the interleaving of similar but not identical topics during the learning process (Rohrer, 2012), and the spacing of a given learning time across more than one learning session, which is also called distributed practice (Cepeda et al., 2006).

Most of these learning strategies have been studied extensively in the laboratory and are known to result in robust and strong performance improvements as compared to the respective control conditions (Bertsch et al., 2007; Rohrer, 2012; Rowland, 2014; Adesope et al., 2017; Brunmair and Richter, 2018). However, less is known about the effects of these strategies in real learning contexts because field studies are relatively rare so far. Moreover, it is still an open question to which degree learning strategies related to desirable difficulties are used in the context of self-regulated learning and which learner characteristics are associated with their use. The present study addresses this question by examining the effect of distributed practice as a learning strategy in a real learning context involving self-regulated mathematical learning of high school students.

### Distributed Practice in Mathematical Learning and in School

Distributed practice means that a given learning or practice duration is distributed across more than one learning session, whereas in massed learning, the same time is spent in one learning session only (Carpenter et al., 2012). The effect of distributed practice has been explored for decades in numerous studies, which suggest robust and large effects on long-term performance in particular, as compared to massed practice (Cepeda et al., 2006). The effect of distributed practice has been demonstrated under a great range of circumstances: with different materials (e.g., Carpenter et al., 2012), in different age groups (e.g., Toppino et al., 1991), and using different lags between the learning sessions (e.g., Cepeda et al., 2009).

However, there are still contexts and conditions that have been considered less so far. These include the effect of distributed practice on mathematical learning in general and, in particular, on mathematical learning in school. An exception are the studies by Rohrer and Taylor (2006, 2007) on distributed practice of mathematical procedures in college. The students in these studies practiced permutation problems either massed in one session or distributed across two sessions with a lag of 7 days. The number of problems that were practiced and the total practice time were the same in each condition, yet students of the distributed practice condition outperformed students of the massed practice condition 1 week after practice (Rohrer and Taylor, 2007) as well as 4 weeks after practice (Rohrer and Taylor, 2006). Although in these studies the effect of distributed practice was investigated with material other than wordlists – which were often used in the classical studies (e.g., Cepeda et al., 2006) – the learning material was still rather narrow in content (i.e., the application of only one specific formula was practiced) and not very complex (i.e., the correct procedure was learned by heart and not explained to the participants).

Another branch of studies investigated a combination of interleaved and distributed practice, termed “mixed review,” in school (Saxon, 1982; Yazdani and Zebrowski, 2006) and in college (Hirsch et al., 1982). This means that in every practice situation, all topics that have previously been taught are practiced and not only the topic that was just covered. Thereby, practice is distributed across several sessions, and different topics are covered in an interleaved (but accumulating) manner. Mixing exercises on current and previous topics improved performance more than only reviewing the most recent topic in each session (for a review on “mixed review” in mathematical learning, see Rohrer, 2009). In addition, Hirsch et al. (1982) and Saxon (1982) found that the positive effect on mathematics performance was particularly large for students in lower to medium performance ranges.

Only few studies have investigated the isolated effect of distributed practice on mathematics learning in school: Schutte et al. (2015) asked third graders to practice basic addition problems 4 min each day for nearly 3 weeks. Students who distributed their 4 min practice time across the day (1-min practice session four times a day or 2-min practice sessions two times a day) outperformed students who practiced massed each day for 4 min. However, because all students practiced each day for nearly 3 weeks, even those of the “massed practice” condition where technically practicing in a distributed manner. In another study by Chen et al. (2017), students of Grades 4 and 5 practiced different mathematical topics of their regular curriculum either massed in one session or distributed across three sessions on three consecutive days. The results indicated that distributing practice across 3 days significantly improved performance as compared to one massed practice session. However, generalizations based on these results are limited because the lag between the last practice session and the test was not the same for distributed and massed practicing students. Furthermore, in a study by Barzagar Nazari and Ebersbach (2018), strong evidence for a positive effect of distributed practice 1 week after the last practice session was found for third graders who practiced semi-formal multiplication in three sessions distributed across three consecutive days, as compared to students who worked the same number of practice exercises massed in one session. Five weeks later, however, this effect disappeared. The reason for this vanishing effect might have been that between the test sessions the students practiced content that was related to the study topic in their regular classes, thereby obliterating differences between the practice conditions. In a similar study conducted in Grade 7 with students practicing stochastics, a topic that is more dissociated from other mathematical topics, evidence for a strong effect of practice condition on the final performance was revealed, both 1 and 6 weeks after the last practice session (Barzagar Nazari and Ebersbach, 2018). In another study, comparing the effects of distributed and massed practice of stochastics exercises that was conducted in Grade 7, no evidence for an effect of practice condition was found in the first test 2 weeks after the last practice. However, a later test conducted 6 weeks after the last practice again revealed strong evidence for a positive effect of distributed practice (Barzagar Nazari and Ebersbach, unpublished). An additional, exploratory result of this second study in Grade 7 indicated that the positive effect of distributed practice occurs especially for students in the medium performance range (similar to the results of Hirsch et al., 1982; Saxon, 1982).

In sum, although only few studies have investigated the isolated effect of distributed practice on mathematics learning in school, most findings suggest a positive impact. However, in previous studies, sessions including distributed practice were highly structured by the experimenters or teachers and took place solely in the classroom. In real-world learning settings, especially older students at school and students at university have to do a lot of learning and practice outside the classroom in a more self-regulated manner. The aim of the present study was to investigate whether distributed practice can also be implemented using online exercises in order to improve mathematical performance in a real-world learning context, in which practice relies more heavily on self-regulation. In addition, we examined which learners actually followed the distributed practice schedule.

### Differential Effects of Distributed Practice

One question that has hardly been investigated in studies examining the effects of desirable difficulties in general, and of distributed practice in particular, is whether all learners profit from such learning strategies in a similar way or whether individual differences moderate the effectivity of distributed practice (Delaney et al., 2010). However, this is a central question in order to decide whether distributed practice can be recommended in general for educational contexts or only for particular contexts or learners. Previous studies described earlier showed larger effects of distributed practice for students with low or medium prior knowledge (Hirsch et al., 1982; Saxon, 1982; Barzagar Nazari and Ebersbach, unpublished). In the two previous studies by Barzagar Nazari and Ebersbach (2018), Barzagar Nazari and Ebersbach (unpublished), several other motivational (e.g., mathematical self-efficacy) and cognitive learner characteristics (e.g., concentration difficulty) were also considered as potential moderators of the effect of distributed practice. Except for the abovementioned baseline performance, no interactions with the effect of distributed practice on test performance were found. However, the sample sizes in these studies might have been too small to reveal moderator effects.

As noted previously, most of the aforementioned studies employed a highly teacher-guided practice procedure. In a more self-regulated learning scenario, the question of whether and how individual learner characteristics affect the use and effectivity of distributed practice actually becomes even more important: Individual motivational and cognitive traits could not only affect the effect of distributed practice on final test performance, but also determine if and how the students follow the respective practice schedule. Distributed practice requires learners to repeatedly engage with a topic or procedure, which may be difficult to retrieve given the temporal delay between learning sessions. In fact, this is why distributed practice is related to desirable difficulties: It is assumed that the lags between practice sessions make the learning process more difficult, which in turn should improve long-term retention. Learners with low mathematical self-efficacy, however, could suffer from this additional difficulty and decide to stop to engage mentally or in practice with the topic (Zimmerman, 1995). That is, the effect of distributed practice and/or the amount of practice may be smaller for learners with low mathematical self-efficacy. A similar reasoning can be applied to performance avoidance goals (Elliot, 1999; Dalbert and Radant, 2008), because students who have problems coping with their mistakes may have relatively more problems with distributed practice, given that the distributed practice schedule initially increases the number of mistakes. Concerning work avoidance (Nicholls et al., 1990) different scenarios are possible: On the one hand, massed practice requires students to work for a longer duration at a time, which might be disfavored by students with high work avoidance. On the other hand, in distributed practice students have to repeatedly bring themselves to start working, which might also be hard for students high in work avoidance. That is, work avoidance possibly could influence the effect of distributed practice and/or adherence to the practice schedule in different ways. Students with low concentration ability, however, might particularly benefit from distributed practice, as the distributed sessions are shorter than one massed session and hence requires the students to concentrate for a shorter duration at a time. Because there is currently only little prior research on the effects of (mathematical) self-efficacy, performance avoidance goals, work avoidance, and concentration difficulty on the efficacy of distributed practice, the interactions of these four characteristics with the distributed practice condition will be investigated in exploratory analyses, with a focus on the students’ adherence to the practice schedule within their self-regulated learning^1^.

### Research Question and Hypothesis

The objectives of the present study were to investigate distributed practice in a real learning context including a relatively high degree of self-regulated learning. The main questions were whether distributed practice is used reliably by learners, and which learner characteristics promote (or hinder) its use. The sample consisted of high school students, and the material was relevant for their mathematics curriculum. We expected that distributed practice might not consistently be applied by the students in the context of their self-regulated learning (see also Dunlosky et al., 2013 for similar findings for adults). In addition, individual learner characteristics that might have affected the implementation of distributed practice were analyzed exploratory.

## Materials and Methods

### Ethics Statement

This study was carried out in accordance with the recommendations of the ethics committee of the Faculty of Human Sciences of the University of Kassel and with written informed consent from all legal guardians of the subjects in accordance with the Declaration of Helsinki.

### Participants

In total, 158 students of eight courses from Grades 10 and 11 (first year of senior classes), attending three schools, were requested to participate in the current study. These students were enrolled either in regular math courses or in intensive math courses, depending on their own choice. All schools were located around a medium-sized German city in neighborhoods with inhabitants of a medium socio-economic status. Participation was voluntary and could be terminated at any time. Only students who had written consent from their parents could participate. They were told that they would receive 10 Euro if they completed the study. Signing up for the study required providing an e-mail address, because the experimental part of the study took place online. Of the 87 students who signed up (40 female, 47 male; 58 of regular math courses, 29 of intensive math courses; *M*_age_ = 16 years 5 months, age range: 15–17 years), 43 terminated their participation ahead of time, and only 44 students finished it completely (25 female, 19 male; *M*_age_ = 16 years 6 months, age range: 15–17 years).

### Design

The independent variable was practice condition with two between-subjects levels: One group of students worked the exercises massed in one session and the other group worked the same exercises distributed across three sessions. Both conditions worked a total number of twelve practice exercises (three sets with four exercises each). An expanding interval schedule was used for the practice sessions of the students in the distributed practice condition (see Procedure; Küpper-Tetzel et al., 2014). The dependent variable was test performance, assessed 2 weeks after completing the last practice exercise.

Of the 87 students who initially signed up, 49 were assigned to the distributed practice condition (25 female, 24 male; *M*_age_ = 16 years 5 months, age range: 15–17 years) and 38 were assigned to the massed practice condition (15 female, 23 male; *M*_age_ = 16 years 6 months, age range: 15–17 years). A slightly larger proportion of students was assigned to distributed practice as we expected a larger dropout in this condition. To ensure that the overall math performance level was roughly equal in both practice condition groups before the manipulation, students who signed up for the study were ranked by their most recent mathematics grade and then, within each class and grade level, randomly assigned to one of the two practice conditions. In order to minimize potential effects of class, the ratio of massed and distributed practicing students was similar in each class. As mentioned above, in total only 44 students completed the study (i.e., 17 distributed practicing students with a median math grade of 2.0 and 27 massed practicing students with a median math grade of 2.3^2^).

Additionally, a questionnaire assessing some motivational and cognitive characteristics of the students (see Table 1 for information on the scales and their reliability based on our data) was included. Finally, each time after students had finished an exercise, they were asked to rate how difficult they considered the respective exercise. These questions were included to examine if students of the distributed practice condition did in fact perceive the exercises to be more difficult than students of the massed practice condition and, thereby, if distributed practice proved to be a difficulty as perceived by the learners.

**Table 1:** Instruments used to assess potential moderators.

Motivational characteristics	Employed instrument	Reliability
Mathematical self-efficacy	Seven items of a German *Academic Self-Efficacy Scale for School Children* (Jerusalem and Satow, 1999), adapted to mathematics (Sample item: “In math, I can solve even the difficult problems if I try hard.”)	α = 0.88
Performance avoidance goals	Eight-item German *SELLMO* (Spinath et al., 2012), adapted to mathematics (Sample item: “In math, my main concern is to avoid that the other students think that I am stupid.”)	α = 0.86
Work avoidance	Eight-item German *SELLMO* (Spinath et al., 2012), adapted to mathematics (Sample item: “In math, my main concern is not to have any difficult tests or work.”)	α = 0.85

**Cognitive characteristic**		

Concentration difficulty	Six items of the German *Learning Strategies in College – LIST* (Boerner et al., 2005; Wild and Schiefele, 1994) (Sample item: “When I’m studying, I’m easily distracted.”)	α = 0.92

### Material

In the course of the experiment, the students were introduced to basic statistics by student assistants with teaching experience, who were supervised by the authors. More specifically, the students were taught the definition of variables and their manifestations, the law of large numbers, the sum rule and the interpretation and creation of diagrams. The topic of basic statistics is generally part of the following school year, thus, no class had covered the topic in their current school year prior to the study. The lessons and practice material were prepared with the support of didactics experts with teaching experience in order to make the learning environment as realistic as possible. The complete material (lesson scripts, practice and test sets, and the scoring scheme) is provided in German online ^3^.

Each practice set for the students consisted of four exercises and involved calculating absolute from relative frequencies, using the sum rule for probability calculation, naming variables and values, and preparing calculations for a diagram. The practice sets contained conceptually similar but not identical exercises, that is, solutions could not be learned by heart. Each practice set could easily be finished in less than 30 min. An example of a practice set can be found in the Appendix.

### Procedure

The students were asked to work through all of the practice and test exercises that followed the lecture at home; only the questionnaire and the lecture sessions at the beginning of the study were completed at school. Practicing at home resembles real-world learning settings in that students usually have to do their homework outside the classroom in a self-regulated manner. In order to avoid students being particularly prepared or relying on help for the test at home, the test sheet was announced as “further exercises.”

#### Prior Testing and Introductory Lesson

Prior to the experimental manipulation, the study started with a survey in school assessing students’ mathematical self-efficacy, self-rated difficulty to concentrate, performance avoidance goals and work avoidance. The questionnaire was programmed with LimeSurvey (LimeSurvey Project Team and Schmitz, 2012) and answered individually by the students on tablets that were provided by the survey team. After the students had finished the questionnaire, they worked on a pretest on basic statistics and probability calculation, assessing whether the prior knowledge of students with regard to the study topic was comparable in all conditions. The survey and pretest were followed by three 45 min regular lecture sessions, in which the students were introduced to the topics specified above. The lecture sessions were spread over 2 or 3 days within 1 week, depending on the schedule of the respective class. At the end of the last lecture session, the students were told that, with their parents’ consent, they could voluntarily participate in an online study on the lectured topic if they provided us with an e-mail address, and that they would receive 10 Euro for the completion of the study.

#### Practice and Testing

Between 5 and 7 days after the last lesson, the students received their first practice exercises, provided via a personalized link that was sent by e-mail. The students were not allowed to keep the lesson material, that is, the material was not available for the practice exercises. However, after a practice exercise was completed, the correct solution was displayed on the screen. The test set was similar to the practice sets, but no correct solutions were provided after test exercises. It was not possible to go back to previous pages at any time. The practice and test sheets were created and distributed with the research tool formr (Arslan and Tata, 2017). In the massed practice condition, the students received three practice sets on the first practice day. In the distributed practice condition, the students received the same three practice sets, but only one practice set on the first day of practice, the second practice set 2 days later, and the third practice set another 5 days later (i.e., expanding interval schedule). After the students had received each link, they had one and a half days to finish the exercises provided via that link. This relatively long period resembles classical homework settings and was provided to ensure that the students had enough time to actually work the exercises. However, checking the time spent on the exercises revealed that most students completed the exercises within 1 day. Only few students opened the link on one day and finished the exercises the next day, and even among these students, some may only have clicked on the link without actually starting with the exercises on the first day. That is, the probability that students of the massed practice condition distributed their exercises across one and a half days instead of completing them in 1 day is negligibly small. Moreover, this interval would still have been much shorter than the intervals between the practice sets in the distributed practice condition. Retention performance was tested 2 weeks after the last practice set was completed with exercises that were similar to the practice exercises.

#### Scoring

Each given answer (mostly numbers or single words) was either correct or wrong, that is, no partial points were granted. For each practice and test set, the maximum score was 15 points. Two raters scored the answers independently from each other according to a predefined scheme. Afterward, the scores of both raters were compared (Cohen’s Kappa = 0.92) and differences were discussed and resolved by these raters. To ensure the reliability of the final rating, a third rater rated the answers independently as well. The final ratings of the first two raters were nearly identical to the third (control) rater (Cohen’s Kappa = 0.96). Therefore, the final scores of the two first raters were analyzed.

### Data Analysis

Because of the severe dropout in the course of the study (of 158 eligible students, only 44 finished the study), analyses concerning the effect of practice condition on retention performance turned out to be rather inappropriate: First, the remaining groups were rather small and not of equal size, and second, there seemed to be a selection bias concerning the dropouts, because the rate of completion was much higher in the massed practice condition (71%) than in the distributed practice condition (35%). We nevertheless report the analysis concerning the effect of practice condition for the sake of completeness, keeping in mind these limitations and that the results should be interpreted with caution. We used a Bayesian linear regression model to analyze the test performance, among other reasons because of the particularly small resulting sample size. One advantage of Bayesian modeling is that it provides a range of possible values for each estimated parameter and assigns probabilities to them, which facilitates interpretation especially when the results are not conclusive in classical statistical modeling (Kruschke, 2015). The linear regression model was estimated in R (R Core Team, 2016) using the package brms (Bürkner, 2017). Further R-packages we used for data preparation and analysis were (in alphabetical order): BayesFactor (Morey and Rouder, 2015), partykit (Hothorn and Zeileis, 2015), psych (Revelle, 2016), rstan (Stan Development Team, 2018), and tidyverse (Wickham, 2017).

As the main aim of the study was to investigate whether distributed practice works in self-regulated learning, subsequently exploratory analyses were conducted to examine which students completed the exercises in the context of their self-regulated learning. These analyses address two other questions that are important when implementing distributed practice in school, besides its general effect: (a) Which students are in general willing to invest additional effort into their mathematics learning by signing up for such a study, and, more specifically, (b) which students actually complete the distributed practice condition? On that account, conditional inference tree models were calculated (Hothorn et al., 2015). These models can be assigned to exploratory data mining, which has been frequently used in social and behavioral science (e.g., Salis et al., 2014; for an overview, see McArdle and Ritschard, 2013), and are useful for exploratory data analyses when there are no specific expectations regarding the relationship between a dependent variable and one or more independent variables. In addition, previous problems of overfitting and biases on the variable selection have been overcome in the current models (Hothorn et al., 2006; Strobl et al., 2009).

Conditional inference tree models seek to identify independent variables that can be used to split up the respective sample into groups that are maximally different with regard to the dependent variable (e.g., test performance score or participation in the study). This is accomplished by recursive binary partitioning: First, the model checks if the distribution of the dependent variable is unrelated to all independent variables. If this null hypothesis can be rejected, the model selects the independent variable that has the strongest relationship with the dependent variable (e.g., work avoidance). The sample then is split into two groups, based on the selected independent variable, in a way that minimizes the *p*-value. The resulting two groups (in the example, these could be participants with average or above-average work avoidance and participants with below-average work avoidance) show maximally different distributions of the dependent variable (i.e., low and high test performance). This process is then reiterated for each of the resulting subgroups until the null hypothesis in the first step cannot be rejected any longer.

## Results^4^

First, in the Bayesian linear regression model mentioned above, test performance served as dependent variable and practice condition (distributed vs. massed practice) and performance in the first practice set (sum score) as independent variables. No priors were specified, that is, an improper flat distribution over the reals was used as prior distribution, which means that the results were highly data-driven and hardly influenced by the priors (Bürkner, 2017). The model was checked for proper chain conversion and autocorrelation, indicating no problems in this regard. The mean for the posterior distribution for the effect of distributed practice was about -1. That is, the students of the distributed practice condition were estimated to have a performance about 1 point (out of 15) lower than students of the massed practice condition (95% credible interval = -3.1 to 1.1). The evidence ratio of 0.13 confirms that a negative effect of distributed practice – contrary to our hypothesis – is more likely than no effect or a positive effect (which would be indicated by an evidence ratio of 1 or higher). According to Lee and Wagenmakers (2013), this is moderate evidence for a negative effect of distributed practice – but, again, these results have to be considered with caution.

Because participation in the current study was voluntary, a first conditional inference tree model was performed with the *enrollment in the study* as dependent variable (two levels: enrolled versus not enrolled in the study) in order to investigate which students were willing at all to enhance their math performance in the context of this study. As independent variables, the most recent math grade, the level of the attended math course (two levels: intensive math course versus regular math course), gender, and the cognitive and motivational characteristics listed above were included. The resulting conditional inference tree revealed that the sample could be divided into two groups, depending on whether their initial math grade was equal to/above average or below average (the median of the math grades was 2.7 on a scale from 1: very good, to 6: inadequate). A significantly larger proportion of students with better math grades signed up for the study, compared to students with lower math grades (*p* < 0.001, see also Table 2). None of the other cognitive and motivational variables predicted students’ enrollment in the study.

**Table 2:** Proportion of students who enrolled in the study, separated by the level of their last math grade.

	Enrollment	
Last math grade	Yes	No
Median or above	69% (*n* = 61)	31% (*n* = 28)
Below median	38% (*n* = 24)	62% (*n* = 39)

A second conditional inference tree model was performed with the *completion of the study* as dependent variable (two levels: study not completed versus study completed) because much less students completed the study than initially enrolled in the study. The independent variables were the same as in the first model, with two additional variables: condition (two levels, massed practice versus distributed practice) and performance in the first practice set (baseline performance, sum score). Both of these variables were irrelevant at the time of enrollment, because at that point the students were not yet assigned to a practice condition and had not yet completed a practice set (that is, these variables only apply to those who enrolled in the study). For this reason, these two variables were only included in the second model. The resulting conditional inference tree confirmed that the proportion of students who completed all practice sets and the test was significantly larger in the massed practice condition than in the distributed practice condition (*p* = 0.007). Additionally, within the distributed practice condition, a significantly larger proportion of female students completed the exercises compared to male students (*p* = 0.001). For the female students within the distributed practice condition, concentration difficulty had a significant impact on the completion of the study, with a significantly larger proportion of female students with lower concentration difficulty having completed all sets than female students with higher concentration difficulty (*p* = 0.009, see also Table 3). No such differential effects were revealed in the massed practice condition or for male students. None of the other cognitive and motivational variables predicted students’ completion of the study.

**Table 3:** Proportion of students who finished the study, of those who initially enrolled, separated by the statistically relevant variables.

	Study completed	
Practice condition, gender, and self-rated concentration difficulty	Yes	No
Massed practice	71% (*n* = 27)	29% (*n* = 11)
**Distributed practice**		
Female students	60% (*n* = 15)	40% (*n* = 10)
Concentration difficulty	1.69 (0.7)	3.01 (1.1)
Male students	8% (*n* = 2)	92% (*n* = 22)

*Concentration difficulty: Mean (SD in parentheses) based on the females in the distributed practice condition only; range: 0–5 points; higher values correspond to higher concentration difficulty.*

Finally, the mean perceived practice difficulty regarding the practice sets was analyzed using Bayesian *t*-tests, in order to determine whether distributed practice was experienced as being more difficult than massed practice. However, no evidence for differences between the conditions regarding the perceived difficulty was revealed for any of the practice sets or the test.

## Discussion

One of the main purposes of the present study was to investigate the effect of distributed practice on the mathematical performance of high school students using curriculum-relevant material. However, due to a severe dropout rate over the course of the study as a consequence of the study relying on self-regulated-learning, the effect of practice condition on final test performance has a low validity. In the sample that could be analyzed, however, the effect of practice condition unexpectedly indicated a negative effect of distributed practice as compared to massed practice. The main focus, then, was on exploratory analyses that were performed to identify factors that contributed to the students’ participation in the study and completion of the study exercises. This is especially important with regard to self-regulated learning, as these factors can give insight into the question of whether students are willing to implement distributed practice in their own learning schedule and, more specifically, which students are willing to do so.

These exploratory analyses revealed some interesting results. First of all, the proportion of students who finished the study was significantly higher in the massed practice condition than in the distributed practice condition. Furthermore, within the distributed practice condition, additional differential effects were found: The proportion of students who finished their exercises was significantly higher among female students than among male students. In addition, within female students who practiced in a distributed manner, the proportion of students who finished the study was significantly higher for girls with low concentration difficulty than for girls with high concentration difficulty. None of these differential effects were found for the massed practice condition. That is, not only did the students complete their exercises more often in the massed practice condition, but for the distributed practicing students, personal characteristics had an additional influence on the completion of the exercises. Taken together, these results imply that distributed practice in self-regulated learning, contrary to massed practice, favors specific students in terms of their willingness to realize this strategy, while others are at a disadvantage. Finally, the perceived difficulty of the exercises was compared between the groups, but there was no difference regarding the difficulty judgments between the distributed and massed practicing students.

Despite the exploratory character of the present results, the differential effects on exercise completion – which were found only within the distributed practice condition – are relevant when implementing distributed practice in school learning. The observed differences between the massed and distributed practice conditions concerning the effects of individual characteristics on the completion of the exercises could be explained by different challenges posed by massed and distributed practice. In contrast to massed practice, with distributed practice the students have to actively decide to resume working on the exercises on multiple occasions, instead of being able to just continue working. That is, action has to be initiated more often in distributed practice than in massed practice, potentially resulting in a higher influence of personal characteristics related to study management on the completion of the exercises (Gollwitzer et al., 1990; Achtziger and Gollwitzer, 2007, 2009). Ultimately, this higher challenge could then lead to fewer completed exercises in the distributed practice condition as shown by our results. The relevant individual factors observed in the present study were gender and concentration difficulty. The fact that more girls than boys completed the distributed practice of mathematics tasks seems to be counterintuitive at first glance as girls usually show less interest in mathematics than boys (e.g., Frenzel et al., 2010). Thus, this effect might be due to other variables than interest, which is also suggested by the finding that no such gender difference was revealed in the massed practice condition. What might stimulate girls in particular to follow a distributed practice schedule? One reason could be that girls presumably possess better self-discipline and self-regulation ability than boys, which is necessary to repeatedly initiate the distributed practice process in self-regulated learning (Duckworth and Seligman, 2006; Martin, 2011; Weis et al., 2013). In this regard, perseverance and the willingness to invest mental effort might be other promising variables that could explain which students employ distributed practice on a self-regulated basis. In fact, females outperform males on these and other related motivational variables (Neigel et al., 2017).

The finding that among females, lower concentration difficulty was associated with a greater success at completing distributed practice tasks contradicts the assumption that learners with poor concentration ability might profit from distribution. However, concentration ability is associated with a higher engagement in learning in general (Newmann, 1992; Skinner and Belmont, 1993) and might therefore support these females to complete the distributed practice sessions.

The analyses of the perceived difficulty of the practice and test sets provided no evidence for the fact that the perceived difficulty differed between massed and distributed practicing students. However, it should be noted that the students did not explicitly rate the difficulty of the practice strategy but only the difficulty of the exercises. The students were not able to directly compare the alternative practice strategies and, thus, could not rate the relative but only the absolute difficulty. Especially because the exercises generally were not perceived as particularly difficult, potential differences could have been minimized. Additionally, the lack of a meaningful difference could also be due to prior self-selection, because students who perceived the exercises as particularly difficult may have stopped working on them in the course of the study. That is, the question of whether distributed practice is in fact perceived as more difficult than massed practice should ideally be investigated in studies with a within-subjects design.

### Limitations

First of all, the main results of the present study are rather exploratory and hence should be verified by further studies. The question of whether distributed practice generally improves performance in mathematics compared to massed practice, however, should be investigated in studies with less emphasis on self-regulated learning in order to maintain sufficient sample sizes and reduce potential selection bias. Additionally, though the topics were picked from the regular curriculum and hence were generally relevant for the students, their performance in our study did not influence their math grades and was not even shared with the teacher. This could have negatively impacted the motivation to participate in and complete the study. Ideally, in future studies on distributed practice in a self-regulated learning context, the personal relevance of the learned content should be increased compared to the current study – for example, by grading the performance. Finally, the students worked on the exercises at home, that is, the context and state in which the students participated in the study was barely controlled. However, this limitation should apply to both conditions equally and is no explanation for the differences between practice conditions.

## Conclusion

One of the original questions of this study of whether distributed practice improves performance in mathematical learning in high school can hardly be answered based on the present study. The moderate evidence for a negative effect of distributed practice should not be overemphasized due to a high likelihood of self-selection in the course of the study. As long as there is no further empirical confirmation of this unusual result, the general assumption that distributed practice improves performance in later tests compared to massed practice (Carpenter et al., 2012), even with coherent mathematical material (Rohrer and Taylor, 2006, 2007; Schutte et al., 2015), should be maintained. However, it should be seriously questioned whether this advantage holds if students ultimately complete less exercises under distributed practice conditions, as observed in the current study. The main finding here was that – in contrast to massed practice – distributed practice in semi-self-regulated learning (as the schedule was externally given and not chosen by the students themselves) seems to favor students with particular characteristics: in the current study, female students with lower concentration difficulty. Because self-regulated learning plays an important role especially in high school and at university, these differential effects concerning the application of distributed practice may be problematic if they result in performance improvements of a particular group of students while disfavoring others. Teachers may prefer strategies that improve the performance of all students equally. Therefore, it is vital to know whether and which learners are capable of successfully implementing distributed practice into their own learning schedule.

In future research it should be investigated if the implications of the exploratory results of this study can be replicated and whether and how students can be supported by implementing distributed practice effectively in their self-regulated learning. A potential measure to motivate students to keep on working even in the distributed practice schedule could be to inform them prior to the practice phase about the positive effects of distributed practice. For example, at least in completely self-regulated learning, Ariel and Karpicke (2018) could show that informing students about the positive effect of retrieval practice resulted in higher use of this strategy. That is, increasing the metacognitive knowledge of students could motivate them to implement even practice strategies that are more challenging, a mechanism that could also help to increase the use of distributed practice. To sum up, assuming that distributed practice – when implemented under external control – improves mathematical performance of learners, the question of whether this advantage emerges only for a subgroup of learners under self-regulated learning conditions is crucial and should be further investigated.

## Data Availability Statement

The datasets and analysis scripts for this study are part of the Supplementary Material repository (osf.io/egt4j).

## Author Contributions

Both authors developed the basic idea of the present study. KBN was responsible for the study design and data collection procedure, conducted the analyses, and wrote a first version of the manuscript, which was then repeatedly revised by both authors.

## Conflict of Interest Statement

The authors declare that the research was conducted in the absence of any commercial or financial relationships that could be construed as a potential conflict of interest.

## Figures and Tables

**FIGURE A1:**
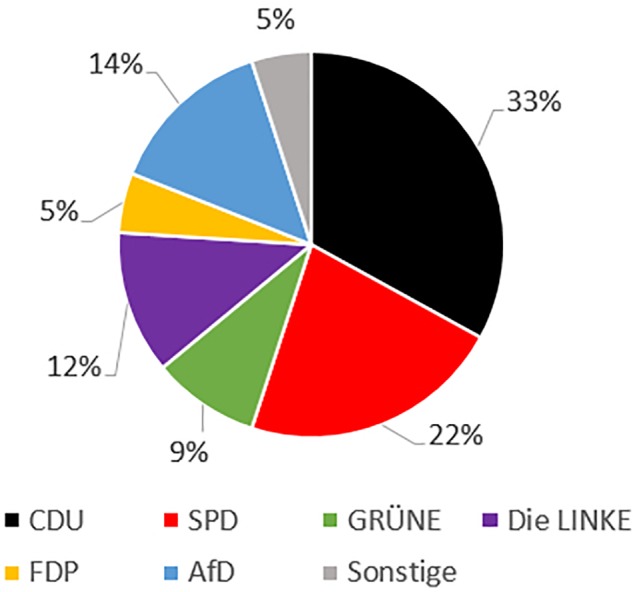
Pie chart for exercise 1.

## Footnotes

1 Originally, another purpose of the present study was to investigate the effect of the practice condition on the test performance and possible interactions with the mentioned characteristics. However, as the study suffered a severe dropout, the final sample was too small to examine interaction effects on the test performance. Therefore, the exploratory analyses are limited to the dependent variables of participation and adherence to the practice schedule (see the section “Results”).

2 In Germany, grades range from 1 (very good) to 6 (inadequate).

3 osf.io/egt4j

4 The data as well as analysis scripts are provided online (osf.io/egt4j).

## Appendix: Example Exercise Set

Each exercise set consisted of four exercises similar to the following. The complete material was provided in German language online (osf.io/egt4j).

### Exercise 1 – Political Parties

The so called Sunday question (‘Sonntagsfrage’) is used to determine the political mood in Germany. Last Sunday, 1,300 citizens answered the following question: ‘Which political party would you vote for if there was an election next Sunday?’

How many citizens would vote for the ‘CDU’ and ‘Die Grünen’? How many citizens would vote for the ‘AfD’?

**Solution:**

CDU: 1300/100 = 13 → 13 ^∗^ 33 = 429 voters
Die Grünen: 1300/100 = 13 → 13 ^∗^ 9 = 117 voters
AfD: 1300/100 = 13 → 13 ^∗^ 14 = 182 voters

### Exercise 2 – Dices

The six sides of a quadratic cube are labeled with numbers from 1 to 6. The following probabilities apply to the different sides:

P(1) = P(6) = 0.07
P(2) = P(5) = 0.13
P(3) = P(4) = 0.3

Calculate the probability for the following events if the dice is rolled once:

(a) The dice rolls a 1 or a 6.
(b) The dice rolls an even number.
(c) The dice rolls a 3 or a 5.

**Solution:**

(a) The dice rolls a 1 or a 6: P(1) + P(6) = 0.07 + 0.07 = 0.14
(b) The dice rolls an even number: P(2) + P(4) + P(6) = 0.13 + 0.3 + 0.07 = 0.5
(c) The dice rolls a 3 or a 5: P(3) + P(5) = 0.3 + 0.13 = 0.43

### Exercise 3 – Students I

In the winter term 2015/2016 there were 244.322 students enrolled in different types of universities in Hessen. The distribution was as follows:

**Table A1:** Table for exercises 3 and 4.

University	154274
Theological University	764
University of Arts	1737
University of Applied Science	83411
University of Management	4136

Please indicate the sample, name the characteristic (variable) and the possible values.

**Solution:**

Sample: all students = 244322
Characteristic (variable): type of university
Values: University, Theological University, University of Arts, University of Applied Science, University of Management

### Exercise 4 – Students II

**Table A2:** Table for exercises 3 and 4.

University	154274
Theological University	764
University of Arts	1737
University of Applied Science	83411
University of Management	4136

Calculate the missing relative frequencies and the angles for a pie chart:

University: ___
Theological University: 0.003
University of Arts: 0.007
University of Applied Science: ___
University of Management: ___

University: ___
Theological University: 1.08°
University of Arts: 2.52°
University of Applied Science: ___
University of Management: ___

**Solution:**

Relative frequencies:

University: 154274/244322 = 0.63
Theological University: 0.003
University of Arts: 0.007
University of Applied Science: 83411/244322 = 0.34
University of Management: 4136/244322 = 0.02

Angles:

University: 0.63 ^∗^ 360 = 226.8°
Theological University: 1.08°
University of Arts: 2.52°
University of Applied Science: 0.34 ^∗^ 360 = 122.4°
University of Management: 0.02 ^∗^ 360 = 7.2°
